# Is there a role for novel supplements in the management of fatigue in rheumatic diseases?

**DOI:** 10.1136/rmdopen-2024-004529

**Published:** 2024-07-27

**Authors:** Thomas Khoo, Meghna Jani, Hector Chinoy

**Affiliations:** 1 Faculty of Health and Medical Sciences, The University of Adelaide, Adelaide, South Australia, Australia; 2 Rheumatology Units, Southern Adelaide Local Health Network and Royal Adelaide Hospital, Adelaide, South Australia, Australia; 3 Division of Musculoskeletal and Dermatological Sciences, Faculty of Biology, Medicine and Health, The University of Manchester, Manchester, UK; 4 Department of Rheumatology, Salford Royal Hospital, Northern Care Alliance NHS Foundation Trust, Salford, UK; 5 NIHR Manchester Biomedical Research Centre, Manchester University NHS Foundation Trust, Manchester Academic Health Science Centre, Manchester, UK; 6 Centre for Epidemiology Versus Arthritis, Centre for Musculoskeletal Research, The University of Manchester, Manchester, UK

**Keywords:** Fatigue, Therapeutics, Social Media

## Abstract

Fatigue is a common symptom of rheumatic diseases and frequently persists even when patients are in a near-remission state. In seeking options to manage troublesome symptoms such as fatigue, complementary and alternative medicines (CAM) are often used by patients despite a lack of evidence base behind such treatment strategies. CAM use is further promoted by social media and ‘influencer’ marketing without rigorous process to ensure scientific accuracy. One mechanism of recent interest in the CAM market is enhancing cellular pathways of nicotinamide adenine dinucleotide (NAD+), purported to restore mitochondrial function. However, clinical trials of NAD+ pathway supplements lack rigorous trial design, many declare conflicts of interest, and safety data is limited. Ultimately, CAM use by our patients is unavoidable. To adequately inform patients about CAM, we need to keep updated on both the latest scientific literature and social media trends. In so doing, we can then propose to patients how standard-of-care therapies, evidence-based lifestyle modifications and CAM might safely and effectively integrate to form a treatment plan.

 Rheumatic diseases comprise a heterogeneous group of conditions with varying contributions from autoimmunity, inflammation and mechanical insult. Therapeutic options for rheumatic conditions have diversified over the last few decades, yet patients continue to self-report symptoms that are inadequately addressed, despite the treatment response meeting definitions of so-called remission. The challenge lies in determining whether remission criteria are lacking,^1^ persisting symptoms reflect a difficult-to-treat disease phenotype,^2^ or treatment approaches need further optimisation.

Fatigue remains a particularly troublesome, challenging to manage, and persistent symptom in patients with rheumatic diseases. Even when patients with rheumatoid arthritis achieve a state of near-remission, they still report similar impacts of the disease (eg, pain, impaired well-being, fatigue) compared with a non-remission state.^3^ The mechanisms underlying fatigue in autoimmune diseases are likely to be multifactorial, with one theory being the role of mitochondrial dysfunction in impaired metabolism^4^ and autoimmunity.^5^

Over the last 50 years, there has been growing acceptance of the endosymbiosis theory; that eukaryotic cells arose billions of years ago through the symbiosis of two prokaryotic bacterial cells, one of which became mitochondria within the other.^6^ As a more recent extension of this theory, autoimmunity and inflammation have been proposed to occur partly as a result of the breakdown of endosymbiosis and impaired autophagy, leading to persistent reactive oxygen species, mitochondrial metabolites and nucleic acids.^5^ Failure of endosymbiosis underpins mitochondrial pathology that extends beyond the traditional paradigms of maternally inherited genetic defects. That autoimmune diseases are rising in incidence^7^ may be in part explained by the idea that today’s society is more vulnerable to mitochondrial dysfunction through engendering a culture of sedentary lifestyle, sleep disruption and ultra-processed food consumption.^5^

In 2023, the European Alliance of Associations for Rheumatology (EULAR) published guidelines recommending the regular assessment of patients with rheumatic diseases for fatigue and providing structured management strategies to address fatigue.^8^ Notably, the EULAR guidelines focus on non-pharmacological methods such as psychoeducation and physical therapies. In seeking other options to manage musculoskeletal complaints and take control of their disease, complementary and alternative medicines (CAM) are frequently used by patients despite a lack of evidence base behind such a treatment strategy. CAM comprise a varied group of substances, supplements, therapies and procedures that may contribute to health and well-being but are not part of conventional, standard care. CAM are not subject to the rigorous route that medicinal drugs require for approval including clinical trial evidence, and stringent approval from regulatory agencies. Non-adherence to standard-of-care medications in rheumatology is considerable^9^ and unsurprisingly, the main reasons cited by patients for using CAM include dissatisfaction with conventional medicines, pursuit of more holistic options and the perceived safety of CAM.^10^ However, the evidence base for CAM use is sparse and potential safety considerations such as possible CAM–drug interactions are unknown. Overall, consensus guidelines within rheumatology are needed.^11^

In the present day, the augmented global connectivity enabled through social media and the emergence of ‘influencer’ marketing encourage the promotion of CAM.^12^ Social media is a place where content is not readily assessed for accuracy and where the boundaries between cosmetic products, supplements and medications are indistinct. Anti-ageing, anti-inflammatory and immune-boosting properties of CAM are robustly promoted online.^13^ It is therefore understandable where the appeal for CAM use arises for rheumatology patients with challenging symptoms such as fatigue, which currently has limited therapeutic options.^13^

One pathway that has attracted much interest in the CAM market is the purported restoration of mitochondrial function through the supplementation of nicotinamide adenine dinucleotide (NAD) pathways (figure 1). A decline in NAD+ has been linked to multiple age-related changes in animal models and humans, including genomic instability, mitochondrial dysfunction and telomere attrition.^14^

**Figure 1:**
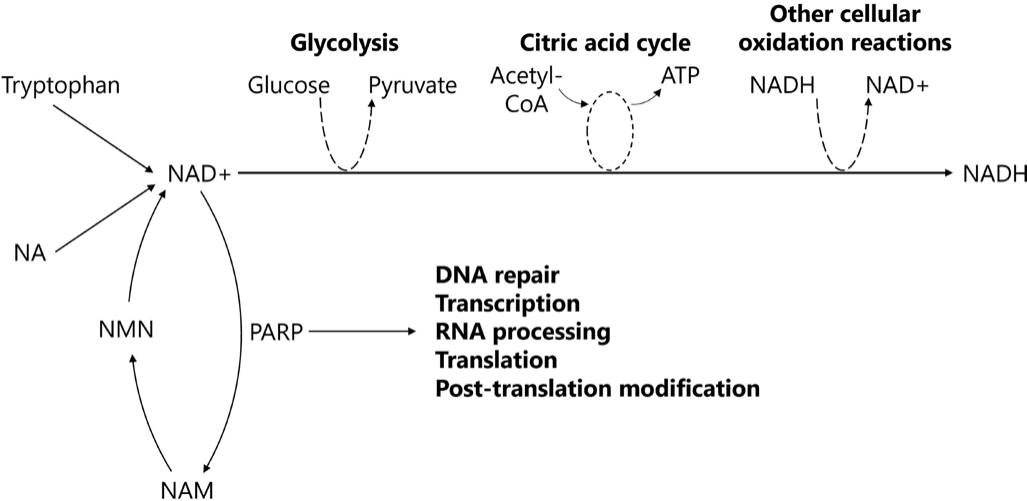
A simplified diagram of the cellular functions of NAD+. NAD+ is synthesised from tryptophan (via the kynurenine pathway), NA (via the Preiss-Handler pathway) and NAM (via the intermediate, NMN, through salvage/recycling pathways). The reduction reaction of NAD+ to NADH is involved in the oxidative steps of glycolysis and the citric acid cycle, as well as the reduction of reactive oxygen species produced from other cellular oxidation reactions. Additionally, NAD+ facilitates PARP, an enzyme involved with DNA repair in the nucleus and protein synthesis in the nucleus and cytoplasm. ATP, adenosine triphosphate; NA, nicotinic acid; NAD, nicotinamide adenine dinucleotide; NADH, nicotinamide adenine dinucleotide+hydrogen; NAM, nicotinamide; NMN, nicotinamide mononucleotide; PARP, poly (ADP-ribose) polymerase.

The desired effect of increasing cellular NAD+ to restore mitochondrial function has been proposed through three main mechanisms^15^:

1. Directly supplementing NAD+
2. Supplementing NAD+ precursors (nicotinamide mononucleotide (NMN), nicotinamide riboside (NR), L-tryptophan, nicotinic acid or niacin (NA))
3. Inhibiting NAD+ degradation by CD38 and poly-ADP-ribose polymerases (PARP)

Public interest in NAD+ supplements continues to grow, with consumer market research anticipating accelerating interest this decade.^16^ Trends of increasing supplement use appear to be fuelled by early and generally scant research findings, ageing populations focusing on the pursuit of supplements with anti-ageing properties and the profound influence that social media holds on today’s society. As dietary supplements rather than pharmaceuticals, NAD+ pathway effectors have also become readily available without rigorous experimental evidence supporting their use.

The impact of coupling favourable preclinical research with the ignition of social media promotion is demonstrated by the supplement resveratrol, which is frequently combined with NAD+ precursors such as NMN. Initial promising research into resveratrol, published in *Nature*, demonstrated anti-ageing effects in yeast,^17^ roundworms and *Drosophila* flies,^18^ and protective effects in mice from a high-calorie diet.^19^ At a molecular level, resveratrol activates sirtuins, a group of deacetylase enzymes that require NAD+ as a co-substrate to function.^20^ Consequently, one of the purported benefits of consuming NAD+ and resveratrol supplements together is synergistic mitochondrial activation.^21^

However, extensive efforts to translate resveratrol from in vitro and animal models to human research have largely been unsuccessful and remain controversial.^22^ Clinical trials of resveratrol use for cancer, metabolic syndrome and neurological disorders have ambiguous results with endpoints that do not translate to clinical outcomes.^23^ Nevertheless, resveratrol’s protean benefits continue to be marketed, and now, NAD+ supplements are embarking on a similar trajectory of public interest predicated on limited scientific evidence.

Human trials involving supplements that aim to replete NAD+ have only occurred with small sample sizes and many declare conflicts of interest. Trial outcomes are conflicting, and even though there are reports of self-reported drowsiness improving with NMN supplementation,^24^ the use of unvalidated outcome measures and a lack of rigorous trial design limit the generalisability of results. Although frequently aimed at bolstering mitochondrial function, there is no good evidence that this is achieved through supplementing the NAD+ pathway.^25^ There is some interest in clinical trials of NAD+ supplementation for post-COVID-19 fatigue and long-term COVID-19 syndromes but results, whether favourable or not, have yet to be published.^26^ Limited safety data are available from clinical trials, and very few reports have been made to adverse event reporting databases such as the Food and Drug Administration or the Medicines and Healthcare Products Regulatory Agency. Except for a single 6-month trial, all other trials of NAD+ pathway supplementation have been less than 3 months and so lack long-term safety data.^27^ In a mouse model of adjuvant-induced inflammatory arthritis, NMN supplementation actually exacerbated arthritis severity.^28^

For multifactorial and debilitating symptoms like fatigue, the possibility of an ‘easy fix’ with safe and effective supplements that restore mitochondrial function remains an unrealised dream. As supplements with bioactive molecules, NAD+ pathway CAM require further systematic evaluation of commercially available products for quality, and detailed pharmacokinetic and pharmacodynamic studies. Investigator-initiated studies and unbiased methodologies involving industry collaborations with academia are needed. Large trials and longer-term follow-up for safety signals in cohorts of specific rheumatic conditions, rather than basket design trials in heterogeneous disease groups, would also be helpful.

As physicians, we should be aware that CAM use by our patients is unavoidable. Although a high proportion of rheumatology patients report having tried CAM, there does not appear to be an association between CAM use and a lack of trust in one’s physicians.^29^ Consequently, physicians still have an important role in educating patients about CAM use. From the evidence available, CAM are not disease-modifying and do not alter the natural history of rheumatic diseases, yet pose possible risks: side effects, interactions with standard care medications and financial burden. Although there are freely available online checkers for interactions between prescribed medications, similar resources for CAM are not easily accessible. Therefore, any advice given on CAM should include a non-judgemental discussion that CAM use is non-licenced and supplements affecting the NAD+ pathway cannot be medically recommended. Holistic lifestyle discussions with patients are a prime opportunity to discuss empowering self-management strategies, including smoking cessation, healthy eating/weight loss, regular exercise, sleep hygiene and the impact of stress.

A position of universal caution is arguably not enough. To adequately inform patients on the potential harms and benefits of CAM, as a rheumatology community we are now obliged to keep abreast of both the scientific and the social media literature. Additionally, we know that patients frequently seek out information from multiple sources other than their doctors.^30^ Not everything online is misleading; increasingly, medical blogs and podcasts citing trials of sufficient scientific and statistical rigour are trying to discuss the science (or lack thereof) behind the latest trends. Patient advocacy organisations are key sources of information and are often linked to real-life and virtual support groups. In allying with these advocacy groups, we can work together towards generating consistent, accurate and accessible resources for patients on CAM use. These resources can then be referred to in the clinic, advertised through support groups and distributed via patient-initiated forums. Self-management courses organised for patients in the community are becoming more popular and may be another avenue to provide practical approaches to improving lifestyle factors.

From a mechanistic perspective, there are plausible benefits of enhancing NAD+ pathways. In particular, for treating rheumatic diseases, novel options targeting difficult-to-treat symptoms such as fatigue are appealing. For physicians, proactively and openly asking patients about health messages from external sources such as the Internet allows us to appropriately address questions within the context of prior healthcare experiences, expectations of CAM use and hopes for symptom improvement. By understanding our patients better, helping them to understand themselves better, linking with advocacy groups and supporting community-based initiatives, we can then promote a sensible narrative involving standard-of-care therapies, evidence-based lifestyle modifications and CAM that might optimally achieve disease remission.
